# A zooprophylaxis strategy using l-lactic acid (Abate) to divert host-seeking malaria vectors from human host to treated non-host animals

**DOI:** 10.1186/s12936-020-3136-9

**Published:** 2020-01-30

**Authors:** Elison E. Kemibala, Agenor Mafra-Neto, Teun Dekker, Jesse Saroli, Rodrigo Silva, Anitha Philbert, Kija Nghabi, Leonard E. G. Mboera

**Affiliations:** 1 Ministry of Health, Community Development, Gender, Elderly and Children, Vector Control Training Centre, Muheza, Tanzania; 2 ISCA Technologies, Riverside, USA; 3 University of Dar es Salaam, Dar es Salaam, Tanzania; 4 Swedish University of Agricultural Sciences, Alnarp, Sweden; 5 SACIDS Foundation for One Health, Sokoine University of Agriculture, Morogoro, Tanzania

**Keywords:** Mosquitoes, Malaria, Attractant, l-Lactic acid, Goats, Human host

## Abstract

**Background:**

Zooprophylaxis is a technique in which blood-seeking vectors are diverted to non-host animals in order to lower blood-feeding rates on human hosts. The success of this technique depends on the host preference of the vector being targeted. The objective of this study was to evaluate the effect of l-lactic acid (Abate) to divert malaria mosquito, *Anopheles gambiae* from feeding on human host.

**Methods:**

A 14-month-old female goat was treated with Abate, a formulation incorporating l-lactic acid into a slow-release matrix. This formulation was applied on the fur of the goat’s back and neck. The treated animal was then presented to *Anopheles gambiae* sensu stricto (*s.s*.) as a prospective host in a semi-field environment (‘mosquito sphere’) together with either an untreated animal or a human. The number of mosquitoes caught to each host choice offered were compared.

**Results:**

Goat treated with the l-lactic acid formulation successfully attracted *An. gambiae* at higher rates (70.2%) than the untreated ones (29.8%). Furthermore, *An. gambiae s.s.* were attracted to a treated goat at an equivalent degree (47.3%) as to their preferred human host (52.7%), even when the preferred host was present in the same environment.

**Conclusions:**

The findings indicate that human host-seeking mosquitoes can be diverted into feeding on non-preferred hosts despite the close proximity of their favoured host, hence reducing chances for the transmission of blood-borne parasites.

## Background

Malaria is a mosquito-borne disease placing a huge burden on public health especially in tropical regions of the world. In 2017, malaria caused an estimated 219 million clinical episodes and 435,000 deaths, with the 93% of these deaths occurring in sub-Saharan Africa [1]. The most efficient malaria vectors in sub-Saharan Africa belong to the *Anopheles gambiae* sensu lato (*s.l*.) and *Anopheles funestus* species complexes [2]. The World Health Organization (WHO) currently recommends the use of long-lasting insecticide-treated mosquito nets and indoor residual spraying of insecticides to protect humans from mosquito bites [3]. For the past decade, massive scale-up of these two interventions has resulted in considerable progress in malaria control across sub-Saharan Africa [4]. However, sustainability of these interventions for year-round or long-term community protection is now facing several challenges [5]. These include difficulties in achieving the required coverage rates during hot and dry seasons, deterring mosquito net usage and routine household damage to nets compromising their protective efficacy [6, 7]. Moreover, IRS applications are logistically demanding and economically unsustainable in many malaria-endemic regions [8]. The increasing frequency of resistance of mosquitoes to insecticides used in mosquito nets and indoor residual spraying further threatens sustainable effectiveness of both strategies [9–12].

To address these challenges and achieve effective, sustainable malaria control, there is an urgent need for exploration of novel solutions to supplement the existing interventions [13]. A highly promising approach that has been explored intensively in recent years is the use of the “Attract and Kill” approach [14]. This approach deploys a two-part formulation i.e. a lure which can emulate vertebrate host odours, floral-based sugar sources or other attractant chemicals; and a lethal or incapacitating component. The lure draws the target mosquitoes to the application site, inducing it to contact and/or feed upon the attract and kill formulation and expose themselves to the control agent within. This exposure either kills or debilitates the mosquito, rendering it less capable of flight, feeding or mating [15].

Mosquitoes use olfactory cues such as carbon dioxide to locate and orient themselves to their blood hosts. However, carbon dioxide is not species specific, meaning that mosquitoes would not be able to distinguish between human and other animal hosts based on this cue [16–19]. Several chemical compounds including l-lactic acid, ammonia, 1-octen-3-ol and phenols have been reported as mosquito attractants [20–24]. Though l-lactic acid has been identified as one of the components contained in the skin and breath secretion of mammals, its concentration varies between different groups of mammals. While highly anthropophilic mosquitoes are attracted by higher concentrations of lactic acid, zoophilic ones are repelled by it [17].

Zooprophylaxis has been a key component in environmental management of mosquito-borne diseases [25, 26]. In this technique, mosquitoes are diverted from humans to other mammals thus reducing individuals’ susceptibility to such [26–28]. Therefore, availability of alternative sources of blood meal for parasite-carrying insects together with other protective measures will likely reduce disease transmission rates. This study, explored an approach to target adult mosquitoes using a zooprophylaxis strategy, deploying a lactic acid-based attractant formulation (Abate) a non-commercial product of the US-based ISCA Technologies Inc. designed to emulate the scent profile of human hosts to divert blood-seeking mosquitoes from people to goats. The study was designated to assess the effect of Abate to divert *An. gambiae* sensu stricto (*s.s.*) into feeding on an animal host, even in the presence of its favored human host.

## Methods

### Study area

This study was carried out in Muheza District located in the northeast of Tanzania. This district (5° 13 ′S, 38° 39 ′E; altitude 193 m) is characterized by a humid and warm climate almost throughout the year. The average annual rainfall in Muheza is 1000 mm with two seasonal peaks i.e. a main peak between March and May, and a less pronounced one between November and December. The mean temperature in the area is 26°C. The cooler months are between June and September while the warmer ones are between October and May. The experiments were carried out in an insectary and in mosquito spheres (semi-field environment) at Amani Research Centre of the National Institute for Medical Research.

### Rearing mosquitoes for experiments

*Anopheles gambiae* Kisumu strain, brought from Kenya Medical Research Institute (KEMR) were colonized and maintained in a controlled environment [27 ± 1 °C, 65 ± 5% relative humidity (RH), and at a 12 h:12 h light: dark cycle] at the Amani Research Centre since early 1982. *Anopheles* larvae were reared in plastic trays (20 cm × 30 cm × 10 cm) filled with distilled water in groups of 250 per tray and fed on fish food (Tetramin^R^) once a day. Adults were kept in cages (30 cm × 30 cm × 30 cm) with access to a 10% sucrose solution. To enable reproduction, female mosquitoes were blood-fed on rabbits according to Standard Operating Procedures approved by the Tanzania Medical Research Coordinating Committee. European Community guidelines and standards were followed in rabbit rearing [29].

### Experiment procedures

Four stages of semi-field trials were conducted in the mosquito spheres [22]. Each sphere contained a mud-made and thatched hut large enough to comfortably house two prospective hosts. In this way, mosquitoes’ response to choose a host pair treated with the lactic acid formulation (Abate) or not was assessed. In the first trial, a pair of untreated goats was placed in the sphere in order to establish a baseline *Anopheles* activity in the presence of natural-state, non-preferred animal host. In the second trial, Abate-treated and untreated goats were presented to assess the effect of lactic acid treatment on feeding behaviour of mosquitoes. This was followed by the placement of untreated goat and untreated human in the third trial sphere to demonstrate mosquito blood feeding behaviour when both preferred and non-preferred hosts were present. This trial sphere was used to compare with host choice presented in the fourth and final semi-field study, during which an Abate-treated goat and untreated human were stationed inside the mosquito sphere. Goats used in these trials were both females, weighing about 14 kg each and both had white fur.

Each hut in the mosquito sphere had a wooden frame designed to accommodate two animal hosts. Two rectangular (90 cm × 50 cm × 60 cm) mosquito nets were used to cover each of the wooden frames during the experiment. Hosts were placed underneath the frames in opposite sides and untreated mosquito nets were draped over each host at the start of each experiment. Human host was not confined in the wooden frame rather covered with nets in a seated position inside the hut. The inner net was extended to the ground while the outer one was kept at about 8 cm from the hut’s floor. The mosquito net was arranged with inner and outer layers which allowed testing of comparative level of mosquitoes’ attraction to the presented hosts and limited their contact with the hosts. Meanwhile, hosts were confined within their assigned frames each night and were not shifted between nights. Mosquitoes captured between the two mosquito nets on each frame were collected using a vacuum aspirator hourly for 6 h from 18:00 h by technician worn a coverall to avoid interfering with mosquito’s choice. At the end of each replicate hourly collected mosquitoes were combined for each choice offered and the totals were used for comparisons.

### Attractiveness of *An. gambiae s.s.* to untreated, non-preferred hosts

In this experiment, two untreated female goats of similar colour and size (about 60 cm standing height), were placed in the hut beneath the designed frames. Specifically, each goat was placed in its own frame and a covering mosquito net. At 18:00 hours, a total of 500 *An. gambiae* sensu stricto (*s.s*.) mosquitoes, previously sugar starved for a period of 6 hours, were released into the sphere until 0:00 hours (midnight) when they were captured from each mosquito net for four consecutive nights. The number of mosquitoes captured between the two mosquito nets was compared to identify the differences in attractiveness between the two hosts. This experiment was repeated for four consecutive nights, with each night representing one replicate.

### Attractiveness of *Anopheles gambiae s.s.* to an Abate-treated goat

This experimental setting was identical to the preceding one except that a prototype formulation of Abate was applied on the skin of one of the goats. About 100 g of the formulation mixed with 100 ml of clean tap water was applied on the fur on the goat’s back and neck using a paintbrush. An Abate-treated goat was then placed underneath one of the hut’s mosquito net frames while the untreated goat (of the same size, age and relative health) was placed underneath the second net frame. A total of 200–400 female mosquitoes were released each night for at 18:00 hours in the evening and collected hourly until 0:00 hours. The collection of released mosquitoes continued for five nights consecutively with each night the treated goat was reapplied with Abate.

### Attractiveness of *An. gambiae s.s.* to human bait and Abate-treated goat

In similar manner to the two preceding trials, an untreated human and goat were presented to starved *An. gambiae s.s.* for host selection. The goat was caged inside one of the netting frames while a human sat in the other. A total of 200–400 female starved *An. gambiae s.s.* mosquitoes were released into the mosquito sphere each night for five nights. After five replicates, the difference in attractiveness between human bait and untreated goat was assessed.

In another series of experiments, mosquitoes were left to choose between an Abate-treated goat and a human bait. The goat was confined in the frame and then covered with double mosquito. In the same hut, a human sat covered by a double-layered mosquito net. A total of 400–600 starved female *An. gambiae s.s.* were released into the mosquito sphere during each night for four night. Capturing of mosquitoes began an hour later and continued at hourly intervals until 0:00 hours for four consecutive nights. Application of Abate on goat’s fur was repeated during each night.

### Data analysis

Data were summarized in Microsoft Excel and then transferred to Stata software (StataCorp. 2013) for statistical analysis.

## Results

Throughout the trial, a total of 5700 starved female *An. gambiae s.s.* were released into the mosquito sphere. Of these, 4926 (86.4%) mosquitoes were captured. In the first experiment, a total of 236 and 201 *An. gambiae s.s.* were collected from the nets covering untreated goat 1 and 2, respectively. There was no significant difference between the number of mosquitoes attracted to the two untreated goats (Table 1).

**Table 1:** Number and percentage and confidence intervals (CI) of captured mosquitoes from untreated goats

Day	Goat 1				Goat 2			
	No.	%^a^	Lower CI	Upper CI	No.	%^a^	Lower CI	Upper CI
1	123	28.15	20.20	36.09	108.00	24.71	16.58	32.85
2	104	23.80	15.61	31.98	83.00	18.99	10.55	27.43
3	8	1.83	− 7.46	11.12	10.00	2.29	− 6.98	11.56
4	1	0.23	− 9.14	9.59	0.00	0.00	0.00	0.00
Total	236	54.00	47.65	60.36	201.00	46.00	39.11	52.89

^a^Percent captured is calculated from total captured i.e. captured in goat 1 + goat 2

When comparing the Abate-treated goat to the untreated one (control); 70.24% of mosquitoes were captured from the net covering the treated goat and the remainder were recovered from the untreated goat (Table 2).

**Table 2:** Number, percentage and confidence intervals (CI) of captured mosquitoes on treated goats versus untreated goat

Day	Treated goat				Untreated goat			
	No.	%^a^	Lower CI	Upper CI	No.	%^a^	Lower CI	Upper CI
1	91	6.06	1.16	10.96	31	2.06	− 2.94	7.07
2	126	8.39	3.55	13.23	44	2.93	− 2.05	7.91
3	267	17.78	13.19	22.36	126	8.39	3.55	13.23
4	326	21.70	17.23	26.18	112	7.46	0.00	0.00
5	245	16.31	11.69	20.94	134	8.92	4.09	13.75
Total	1055	70.24	67.48	73.00	447	29.76	25.52	34.00

^a^Percent captured is calculated from total captured i.e. captured in goat 1 + goat 2

When comparing the mosquito responses to the presence of a human and untreated goat; a larger proportion (76.62%) of the captured mosquitoes were caught while attempting to feed on the human host than on the goat (Table 3).

**Table 3:** Number, percentage and confidence intervals (CI) of captured mosquitoes on untreated goat versus human host

Day	Untreated goat				Human			
	No.	%	Lower CI	Upper CI	No.	%	Lower CI	Upper CI
1	121	8.73	3.70	13.76	202	14.57	9.71	19.44
2	45	3.25	− 1.93	8.43	184	13.28	8.37	18.18
3	27	1.95	− 3.27	7.16	201	14.50	9.63	19.37
4	73	5.27	0.14	10.39	241	17.39	0.00	0.00
5	58	4.18	− 0.97	9.34	234	16.88	12.08	21.68
Total	324	23.38	18.77	27.99	1062	76.62	74.08	79.19

**Fig. 1:**
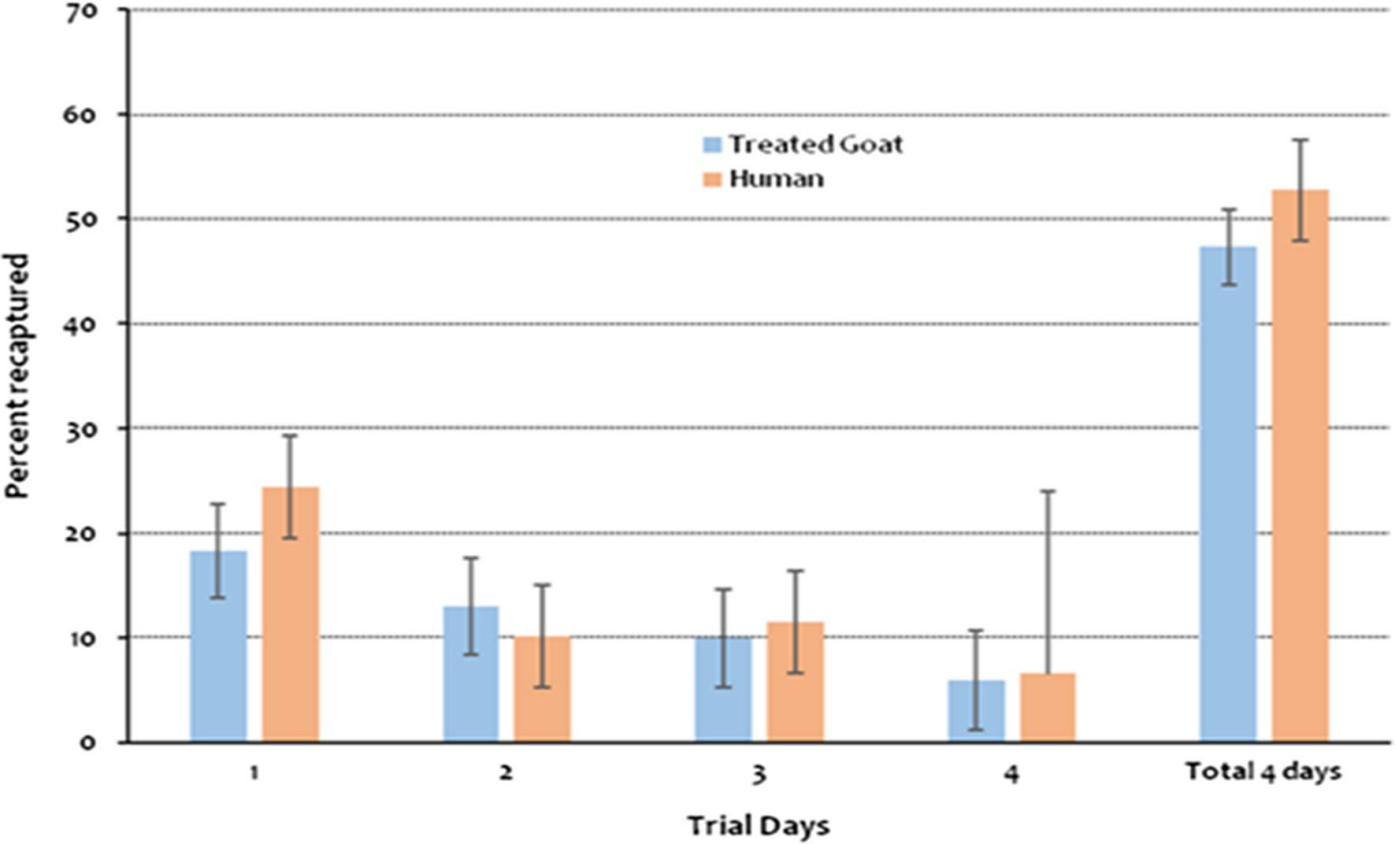
Percentage of collected mosquitoes on Abate-treated goat versus human host

In the trial which compared mosquitoes’ response to human versus to Abate-treated goat; 55% of the captured mosquitoes were caught in the human-baited net while the rest of mosquitoes were from the Abate-treated goat (Fig. 1).

**Fig. 2:**
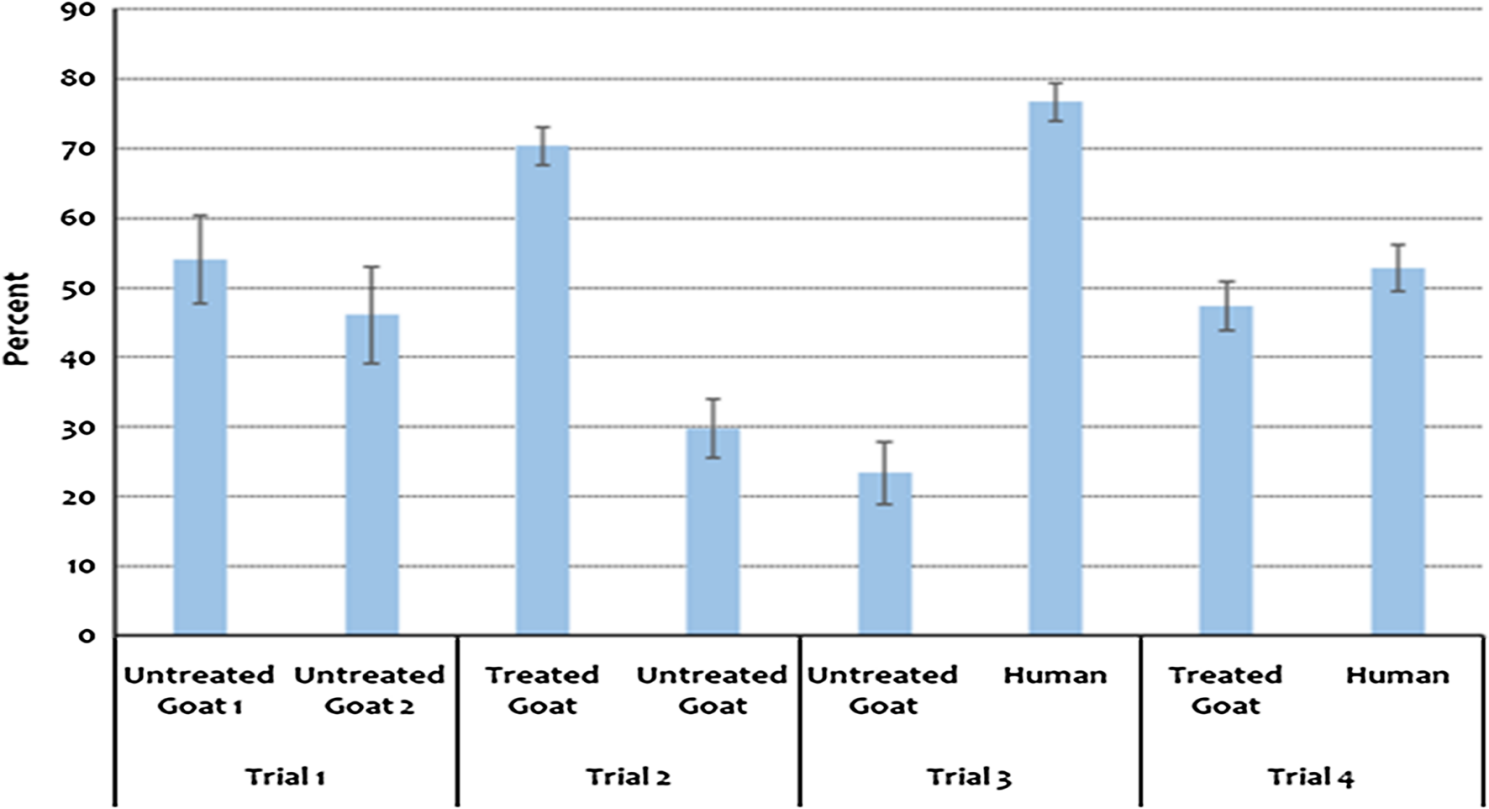
Percentage of mosquitoes collected in the four trials

Generally, the treated goat and human attracted a larger number of mosquitoes during the four trials. In the absence of preferable host, mosquitoes were forced to feed even on non-preferable host. About half (50%) of the mosquitoes fed on non-preferable host in trial 1. On the other hand, less than 30% fed on non-preferred host when the preferred one was present in trials 2 and 3 (Fig. 2).

## Discussion

The findings of this study clearly indicate that Abate formulation is able to attract female *An. gambiae s.s*., and successfully emulated the chemical odour profile of a human host. Topical application of Abate on the furs of treated goat induced *Anopheles* mosquitoes to feed on the treated goat to a nearly equivalent degree of the human host. Generally, the high rate of recapture of released mosquitoes suggest that majority of mosquitoes released into mosquito-sphere were host-seeking and chose one host or the other. The number of captured mosquitoes was much larger in trials involving Abate treatments and humans. *Anopheles gambiae s.s.* mosquitoes are known for their highly anthropophilic tendency in their feeding behaviour [30].

The findings that the number of collected mosquitoes declined from day 1 to 4 indicated that the mosquitoes are likely to have abilities to memorize the previous experience that the real host were not available in their vicinity, and hence reduced their searching activities.

Despite the presence of a human host in the same area, treatment with Abate diverted almost half of the captured mosquitoes into feeding on a non-preferred host. This shows that the use of zooprophylactic strategy in field settings could reduce inoculation rate and chances of malaria transmission to humans. The observed results elucidate that there is a potential in incorporating Abate with a killing agent (such as insecticide) to suppress a large number of mosquitoes in a single setting within a short time. The deployment of such a technique is likely to complement the current malaria mosquito control methods. This attract and kill technique relies not on conventional insecticides but on the synergy of two benign or beneficial components i.e. lactic acid as attractant already produced naturally by the animals themselves (hence, not introducing new component in the environments); and by administering orally based insecticides known to have low toxicity and requiring ingestion of the mosquito in order to work. The latter component provides an opportunity to counteract the challenges associated with contact insecticides. Furthermore, the unusually high levels of lactic acid emanating from Abate-treated animals may dissuade feeding by zoophilic mosquitoes that would otherwise select them as hosts. A previous study found that an artificial addition of non-host cues (lactic acid) to an otherwise suitable animal host often exerts a repellent effect on zoophilic host-seeking insects [17].

Despite the promising results observed in this study, further work is required to optimize the application of Abate formulation in real field settings. One possible potential shortcoming of the current Abate formulation could be the poor persistence of the material on the animal fur when exposed to normal weather during grazing or browsing. Weather conditions like rainfall and sunlight are likely to impact the persistence and effectiveness of Abate application over time [31]. It is equally important to be taken into consideration is how the distance between Abate-treated animals and human hosts to be protected will affect the formulation’s performance. Therefore, further studies focusing on identifying the appropriate killing agent and the characterization of impacts of proximity of human host on the performance of the attractant are required.

## Conclusions

Abate-treated goats were equally attractive to malaria mosquitoes as human hosts. Application of zooprophylactic strategy at the community level will likely reduce the number of mosquitoes encountering and feeding on humans, hence reducing malaria parasite transmission. Future studies should optimize the rate of Abate release and its persistence in different environmental conditions.

## Data Availability

All available data are included in this article.
